# Prediction of 5–year overall survival in cervical cancer patients treated with radical hysterectomy using computational intelligence methods

**DOI:** 10.1186/s12885-017-3806-3

**Published:** 2017-12-12

**Authors:** Bogdan Obrzut, Maciej Kusy, Andrzej Semczuk, Marzanna Obrzut, Jacek Kluska

**Affiliations:** 1 Department of Gynaecology and Obstetrics, Faculty of Medicine, University of Rzeszow, Lwowska 60, Rzeszow, 35-301 Poland; 2 Faculty of Electrical and Computer Engineering, Rzeszow University of Technology, al. Powstancow Warszawy 12, Rzeszow, 35-959 Poland; 3 IIND Department of Gynecology, Lublin Medical University, al. Raclawickie 1, Lublin, 20-059 Poland; 4 Faculty of Medicine, University of Rzeszow, al. Kopisto 2a, Rzeszow, 35-959 Poland

**Keywords:** Cervical cancer, 5–year overall survival, Computational intelligence methods, Probabilistic neural network

## Abstract

**Background:**

Computational intelligence methods, including non-linear classification algorithms, can be used in medical research and practice as a decision making tool. This study aimed to evaluate the usefulness of artificial intelligence models for 5–year overall survival prediction in patients with cervical cancer treated by radical hysterectomy.

**Methods:**

The data set was collected from 102 patients with cervical cancer FIGO stage IA2-IIB, that underwent primary surgical treatment. Twenty-three demographic, tumor-related parameters and selected perioperative data of each patient were collected. The simulations involved six computational intelligence methods: the probabilistic neural network (PNN), multilayer perceptron network, gene expression programming classifier, support vector machines algorithm, radial basis function neural network and k-Means algorithm. The prediction ability of the models was determined based on the accuracy, sensitivity, specificity, as well as the area under the receiver operating characteristic curve. The results of the computational intelligence methods were compared with the results of linear regression analysis as a reference model.

**Results:**

The best results were obtained by the PNN model. This neural network provided very high prediction ability with an accuracy of 0.892 and sensitivity of 0.975. The area under the receiver operating characteristics curve of PNN was also high, 0.818. The outcomes obtained by other classifiers were markedly worse.

**Conclusions:**

The PNN model is an effective tool for predicting 5–year overall survival in cervical cancer patients treated with radical hysterectomy.

## Background

Cervical cancer is the fourth most common malignancy among females worldwide [1]. Approximately 527,600 new cases are reported annually all over the world and 265,700 women died in 2012 [1]. In Poland, the annual incidence of invasive cervical cancer is 8.9/100,000 woman, and in 2012, 2783 new cases of cervical cancer were diagnosed and 1669 women died [2].

The prediction of clinical outcome is a key element of the therapeutic decision-making process. For cervical cancer, the established platform for planning the treatment is the International Federation of Gynecology and Obstetrics (FIGO) classification. Although the FIGO staging system serves as the main tool for estimating the general prognosis, it does not include all established prognostic factors, such as lymph node metastases, lymph-vascular space invasion, deep stromal infiltration, or histologic subtype. Another method for individual prediction of survival in cervical carcinoma is the recently developed nomograms based on selected demographic and clinical parameters [3, 4]. None of these systems take into account intra/postoperative complications and concomitant diseases, however, which could alter patients outcome [5].

The aim of the present study was to develop a universal model for predicting overall survival in individual patients with cervical cancer, based on the demographic characteristics, tumor-related parameters, and selected perioperative data. We used computational intelligence methods, that have been widely applied in oncology [6–10]. Artificial neural networks as advanced computer programs enable the discovery of complex relations within data sets, that cannot be detected with conventional linear statistical analysis. Computational intelligence methods to predict overall survival have not yet been applied for patients with cervical cancer treated by radical hysterectomy.

## Methods

This study originally included 117 patients with cervical cancer (FIGO stages IA2-IIB) treated at the Department of Obstetrics and Gynaecology of the Rzeszow State Hospital in Poland between 1998 and 2001. The preoperative diagnosis was based on the histopathologic examination of tissue material obtained from cervical biopsy and fractionated abrasion. In disputable cases (15 patients), cold knife cervical conization was performed.

All patients underwent radical hysterectomy class III and pelvic lymphadenectomy. Before the surgery, basic laboratory tests, electrocardiograms, and anaesthesiology consultations were performed. Perioperative prophylactic antibiotics and thromboembolic prophylaxis, were applied for each patient. Laparotomy was performed through a vertical midline or low transverse incision, depending on the local conditions. Intraoperative and postoperative complications were recorded and classified prospectively according to the classification proposed by Chassagne et al. [11].

After the period of postoperative recovery, all patients were forwarded to the Department of Gynecological Oncology of the Rzeszow State Hospital. Some of the patients received adjuvant radiotherapy. The qualifying criteria were as follows: presence of metastases to the lymph nodes, lymph-vascular space invasion, or the presence of neoplastic tissue within the surgical incision and non-squamous types of cervical cancer. Radiotherapy was administered in the following manner: teletherapy (50 Gy to the area of pelvis minor in 25 fractions of 2 Gy; BOX technique) and brachytherapy (2 fractions of low-dose rate; total dose 30 Gy). During the study period, chemotherapy was not routinely applied.

Follow-up was conducted once a month during the first year after the operation, every 3 months during the second year of observation, twice annually for 3 to 5 years in the Oncology Outpatient Clinic and the Department of Gynaecological Oncology at the Rzeszow State Hospital, Rzeszow, Poland. The 5–year follow-up data from all the subjects were used to validate examined computational intelligence models designed for the prediction of death within 60 months.

Data available at the time of discharge, derived from histopathologic examination of the surgical specimen and obtained during the follow-up, were collected. In total, 23 variables were identified, including 4 demographic characteristics: age, BMI, hormonal status, presence of concomitant diseases; 13 tumor-related parameters: FIGO stage, histologic type, grade, tumor size ≤4 cm or >4 cm, lymph nodes status, number of lymph nodes dissected, number of positive lymph nodes, lymph node ratio (ratio of positive to totally removed lymph nodes), lymph-vascular space invasion, surgical margins status, parametrial involvement, deep stromal invasion (outer 1/3 of the cervical stroma), postoperative radiotherapy; 6 selected perioperative variables: surgery time, median blood lost, presence of intraoperative complications, presence of postoperative complications, type of complications, and length of hospital stay. The above listed variables are presented in Table 1. To present continuous values, the median measure (along with variable range) was used.

**Table 1:** Demographic characteristics and clinicopathologic data in the study group

Number of patients		102
Median age		46 (29–73)
Median BMI [kg/m^2^]		25.1 (17.5–45.0)
Hormonal status		
	Premenopausal	71
	Postmenopausal	31
Concomitant diseases		
	Hypertension	21
	Diabetes mellitus	3
	Ischaemic heart diseaase	6
	Others	3
FIGO stage		
	IA2	15
	IB1	51
	IB2	8
	IIA	7
	IIB	21
Histologic type		
	Squamous	91
	Non-squamous	11
Grading		
	G1	19
	G2	62
	G3	21
Median surgery time [min]		190 (80–310)
Median blood lost (△Hb) [g%]		3.9 (0.3–7.8)
Tumour size [cm]		
	≤4	69
	>4	33
Median number of removed lymph nodes		13 (1–40)
Lymph nodes status		
	Negative	77
	Positive	25
Median number of positive lymph nodes		0 (0–9)
Median lymph node ratio		0 (0–1)
Lymph-vascular space invasion		
	Absent	83
	Present	19
Deep stromal invasion		
	Absent	66
	Present	36
Parametrium infiltration		
	Absent	78
	Present	24
Surgical margins status		
	Negative	89
	Positive	13
Intraoperative complications		5
Postoperative complications		42
Type of complications		
	Mild	38
	Moderate	2
	Severe	7
Median hospital stay [days]		12 (5–49)
Postoperative radiotherapy		
	Yes	57
	No	45

These variables were used in the simulations, which included six clasiffiers: the probabilistic neural network (PNN), multilayer perceptron network (MLP), gene expression programming classifier (GEP), support vector machines algorithm (SVM), radial basis function neural network (RBFNN) and k–Means method. All considered models were simulated in DTREG software [12].

PNN is a feedforward neural network created by Specht [13]. PNN is composed of the input layer represented by the variables of the input vector, the pattern layer and the summation layer consisting of *G* neurons where each one computes the signal only for patterns that belong to *g*th class. The output layer of the network determines the label for a classified vector in accordance with Bayes’s decision rule based on all the summation layer neuron signals. The performance of PNN can be optimized by selecting the form of the smoothing parameter (*sp*) used for activation of neurons in the pattern layer.

MLP is a feedforward neural network [14]. This network is composed of an input layer, hidden layers, and an output layer. The number of hidden layers, the optimal number of neurons in hidden layers and the appropriate activation functions must be determined for this model.

GEP is an emulating biological evolution algorithm, that creates and evolves computer programs [15]. The programs are encoded by chromosomes composed of the genes. Within the population, evolution is performed by computing the expression of each chromosome, applying predefined genetic operators and calculating the fitness.

SVM is the classification algorithm proposed by Vapnik [16]. The SVM algorithm requires solving the quadratic programming optimization problem. For the SVM model, various kernel functions and their parameters need to be explored. Furthermore, the model’s capacity control parameter *C* must be selected.

RBFNN, similar to PNN and MLP, is a feedforward neural network [17]. This model consists of three layers: an input layer, a radial basis hidden layer, and a linear output layer. The number of neurons in the hidden layer and the parameters of the RBFNN training method must be found [18].

The k–Means clustering algorithm partitions input data into *k* clusters and provides a center of each cluster [19]. As a result, the records within each cluster are similar to each other and distinct from records in other clusters. The predictions for the unknown cases are made by assigning them the category of the nearest cluster center.

The prediction ability of the models was determined based on the accuracy (Acc), sensitivity (Sen), specificity (Spe), and the area under the receiver operating characteristic curve (AUROC). The above parameters were obtained using a 10-fold cross validation procedure [20]. The simulations were conducted 20 times, preserving a random selection of subsets. The results were averaged and the standard deviation was computed. As a reference model, we applied the logistic regression analysis, which is widely used in medical research [10, 21, 22].

## Statistical analysis

The AUROC value of particular classifiers and logistic regression model were compared using pairwise T-tests. Differences were considered statistically significant when *p*<0.05. All statistical analysis were performed using MathWorks’ Matlab R2012a software.

## Results

Among 117 patients that qualified for a radical Piver III hysterectomy and pelvic lymphadenectomy, 15 did not enter the trial: 3 were excluded because the histopathologic analysis of the operative specimen revealed an endometrial cancer with cervical extension, 4 continued postoperative treatment and follow-up at another institution, 3 refused further participation in the study protocol, and 5 were lost from follow-up. The remaining 102 were considered eligible and were enrolled in the study.

The median patients’ age was 46 years (range, 29–73). Thirty-one patients were postmenopausal. The median BMI was 25.1 kg/m^2^ (range, 17.5–45.0). Concomitant diseases were reported in 33 women (Table 1). The prevailing type was squamous-cell carcinoma (89.2%).

The length of surgery ranged between 80 and 310 min (median 190). The median blood loss, measured by a decrease in the hemoglobin level, was △Hb = 3.9 g% (range, 0.3–7.8).

The median number of dissected lymph nodes was 13 (range, 1–40). Positive lymph nodes were diagnosed in 25 patients. The lymph node ratio ranged from 0 to 1. Parametrial involvement was observed in 24 patients, and deep stromal invasion was identified in 36 cases.

Lymph-vascular space invasion was observed in 19 patients. In 13 cases, positive surgical margins were reported. The median hospital stay, calculated from the date of surgery to the day of discharge, was 12 days (range, 5–49).

The number of perioperative complications was 47 (46.1%). Intraoperative complications occurred in 5 patients. Postoperative complications, counted to 30 days after surgery, occured in 42 patients. The vast majority of cases were mild or medium degree complications that did not constitute threats to the health or life of the patients. Severe perioperative complications (pulmonary embolism, bleeding from the inferior vena cava, rupture of duodenal ulcer, genitourinary fistulas) occurred in 7 patients.

Median follow-up period in the study group was 51.7 months (range, 6–60 months). During the follow-up, recurrence was identified in 23 patients (22.6%). Pelvic recurrence was detected in 13 patients. The remaining 10 subjects were diagnosed with distant metastases. The patients’ status at last observation was as follows: alive–79, cancer-related death–23. The overall 5–year survival was 77.5% in the study group.

**Fig. 1:**
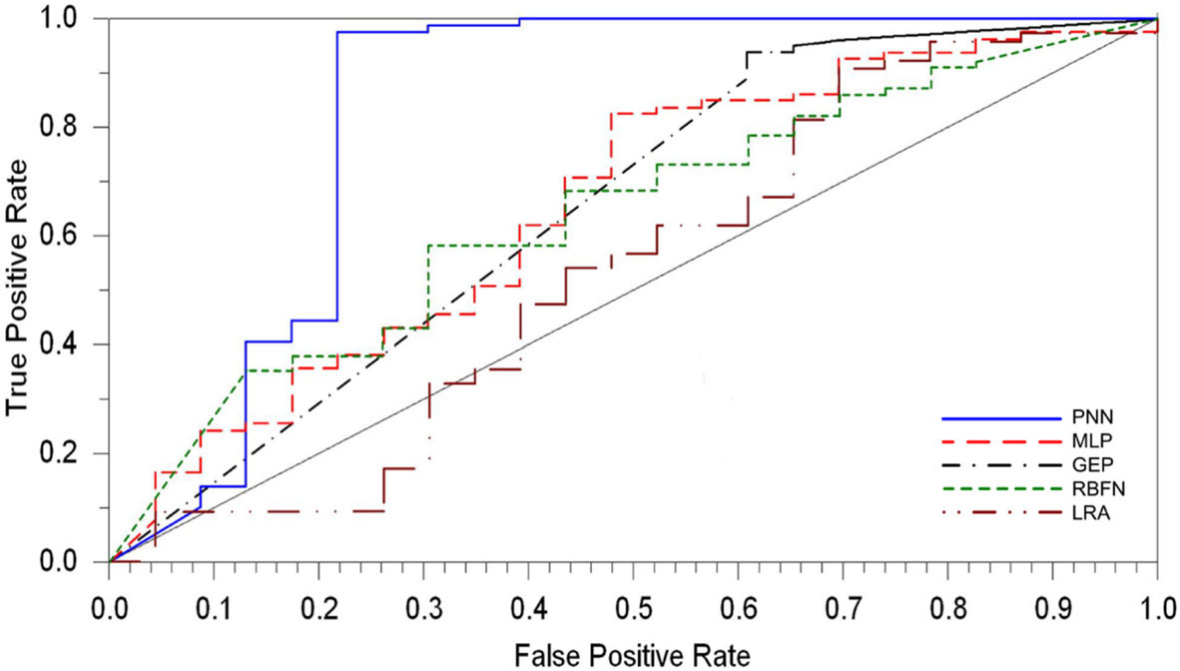
The receiver operating characteristic curves. Plots are shown for the models with AUROC>0.5

The best results in the prediction of 5–year overall survival in cervical cancer patients treated with radical hysterectomy were obtained by PNN. This model enabled the prediction of the 5–year overall survival with the highest accuracy (0.892), sensitivity (0.975), and specificity (0.609). The MLP and GEP also showed high accuracy (0.802 and 0.800, respectively) and sensitivity (≈ 0.93), but markedly lower specificity. The AUROC for PNN (0.818) also significantly surpassed the values of this parameter for the remaining classifiers (Fig. 1). The averaged accuracy, sensitivity, specificity and AUROC value obtained for all applied computational intelligence methods and linear regression model are presented in Table 2.

**Table 2:** The accuracy, sensitivity, specificity and the area under receiver operating characteristic curve obtained for the set of 23 variables

	Acc	Sen	Spe	AUROC	
PNN	0.892	0.975	0.609	0.818	*p*<0.001
MLP	0.802	0.937	0.339	0.659	*p*<0.001
GEP	0.800	0.930	0.352	0.651	*p*<0.001
SVM	0.740	0.956	0.000	0.478	*p*<0.001
LRA	0.703	0.804	0.357	0.559	Non applicable
RBFNN	0.693	0.780	0.396	0.640	*p*<0.001
k-Means	0.611	0.757	0.109	0.406	*p*<0.001

Because PNN provided the highest values of all measured parameters (Table 2), we determined the confusion matrix for this model. As presented in Table 3, of 23 cases of patient death, 9 were incorrectly predicted. On the other hand, only two cases were misclassified among patients who survived.

**Table 3:** Confusion matrix for the PNN model

	Predicted outcome	
Actual outcome	Died	Survived
Died	14	9
Survived	2	77

## Discussion

PNN as well as other computational intelligence methods have been applied to various medical classification tasks [9, 10, 23]. For example, to detect arrhythmia based on digital processing of electrocardiograms [24], for bleeding detection in wireless capsule endoscopy [25], as well as for estimating the risk of mortality after cardiac surgery [26]. In recent years, artificial neural networks have also been used in our studies to predict complications in cervical cancer patients treated by radical hysterectomy [7, 8]. To the best of our knowledge (Pubmed search database), this is the first study to apply computational intelligence methods for prediction of survival in cervical cancer patients treated with radical hysterectomy.

Application of the PNN, which utilized 23 variables (demographic characteristics, tumor-related parameters, and selective perioperative data), enabled the prediction of the 5–year overall survival with an accuracy of 0.892 in cervical cancer patients (Table 2). This model revealed high sensitivity (0.975), although the specificity was lower (0.609). This may result from much lower number of death cases in the study group (class imbalance). Our results are comparable to similar reports in which neural networks were applied for outcome prediction of cancer patients. For example, an artificial neural network was able to predict survival in colorectal cancer patients with an overall accuracy of 90% [6]. The predictive AUROC of PNN model for 5–year survival in esophageal carcinoma was 0.884 [9]. In another study, the ANN model enabled the precise prediction of mortality after primary liver carcinoma with accuracy and AUROC equal to 0.973 and 0.840, respectively [10].

The variables used in the simulations are mostly well-established prognostic factors in uterine cervical cancer. Of these variables, one of the most important is the clinical stage of the disease, as demonstrated in the 1990s [27, 28]. Although the FIGO clinical staging is imperfect, survival rates of patients with cervical cancer correlate with the FIGO stage of neoplasm progression [29].

The correlation between tumor size and prognosis for patients with cervical cancer has also been widely investigated over several decades [30–36]. Some researchers have concluded that tumor size in cervical cancer has an independent prognostic value, regardless of age, ethnicity, histologic grade, or even the type of treatment [37].

Similarly, another known prognostic factor is the depth of stromal invasion of the uterine cervix and infiltration of the parametrium [38–42].

Despite several studies, there is no conclusive evidence regarding the impact of the histologic subtype of cervical cancer on survival rates [43]. While some studies identified no significant differences between cervical squamous cell carcinoma and adenocarcinoma [44–48], others reported unfavorable outcomes for patients with uterine cervix adenocarcinoma [49–53].

Another controversial prognostic factor in patients with cervical cancer is the histologic grade. Some studies indicate that poorly-differentiated squamous cell cervical carcinoma has an unfavorable prognosis [54], whereas another studies did not confirm these observations [55, 56].

Several studies report a close relationship between patient survival and lymph node involvement [57–61]. Moreover, the number of positive lymph nodes is a more accurate prognostic factor than the presence of metastases [62–65]. Attention has recently been focused on another parameter - the lymph node ratio [3].

Factors associated with a higher risk of cancer recurrence include the lymph-vascular space invasion (LVSI). This reflects, in part, the high correlation between LVSI and involvement of the pelvic lymph nodes, yet numerous studies suggest that this parameter is an independent prognostic factor [66, 67].

Published findings related to the potential significance of patient age as a prognostic factor in cervical cancer are contradictory. In several studies, no differences in the survival rates among patients in different age groups were identified [68–71]. Other studies, however, suggest that the prognosis is significantly worse in younger patients [72–74].

A positive surgical margin is considered an important risk factor in the recurrence of cervical cancer. Lee et al. observed a significantly worse disease-free survival and overall survival in patients with positive margins, but only in the univariate analysis [75]. Multivariate analysis showed no significant impact of positive surgical margins on the prognosis. Similar observations were also reported by Landoni et al. [76].

To date, there is no evidence that the surgery time or length of hospital stay have a direct impact on survival. These parameters are related to the course of the operation and convalescence, and can increase due, for example, to complications. Similarly, perioperative complications in themselves do not influence the natural history of carcinoma, but their treatment may delay adjuvant therapy, which in turn, can reduce the probability of survival. Concomitant diseases, reflecting general patient health condition, may also constrain adjuvant therapy and in consequence negatively influence the prognosis [5].

Finally, we used all the listed variables in the simulations because, on one hand, the accuracy of artificial neural networks could be improved by increasing the number of factors [77], and, on the other hand, even non-significant variables must have a non-zero effect on survival [3].

Only a few papers in the literature deal with prediction of survival in cervical cancer patients. Ochi et al. applied artificial neural networks for survival prediction in patients with uterine cervical cancer treated by radiotherapy using different data sets [78]. The highest AUROC value of this model was 0.778. Polterauer et al. developed a nomogram based on six variables: FIGO stage (IB-IV), tumor size (≤ 2 cm vs > 2 cm), age, histologic subtype, lymph node ratio, and parametrial involvement [3]. The value of the c-index, which is conceptually similar to receiver operating characteristics curve analysis [79, 80], was 0.723. The most recent study by Zhou et al. establishes a nomogram predicting 5–year overall survival of surgically-treated stage IA-IIB cervical cancer patients [4]. The authors used a number of metastatic lymph nodes, lymph-vascular space invasion, stromal invasion, parametrial invasion, tumor diameter, and histology as input variables. The c-index of this model was 0.71. A precise comparison of our results with the results presented by others is difficult because of differences in the study population, data set and methodology. Nevertheless, the AUROC value of one of our predictive models (PNN) outperformed the values obtained in the above-mentioned studies.

The presented study has a few limitations. First, the number of patients was too small to enable detailed analysis of the impact of histologic type on survival in the non-squamous cell carcinoma. The group also included adenocarcinomas as well as cases of glandular-squamous, microcellular, and undifferentiated cervical carcinomas. For simulations, we used clinical data from patients treated surgically in 1998–2001. In subsequent years, standards of treatment for cervical cancer were significantly modified. While in past decades, surgery for patients in FIGO stage IIB was quite common, it is currently performed only in highly-specialized centers [81–85]. Moreover, concurrent chemo-radiotherapy has been introduced on a large scale. In this context, the presented results refer to only a selected group of patients.

The current study has also several strengths. The main advantage is its design. As previously mentioned, based on a Pubmed database search, this is the first study to apply computational intelligence methods to predict overall survival in cervical cancer patients treated by radical hysterectomy. Moreover, the study group of patients came entirely from a single institution, where the same principles of diagnostic and surgical procedures were carefully applied. This increases the strength of our study, due to its consistency and uniformity.

## Conclusions

Computational intelligence methods enable credible survival prediction for cervical cancer patients. The prediction ability of the PNN measured by the AUROC value outperformed that of the MLP, GEP and RBFNN and the linear regression model. The low AUROC value for the SVM algorithm and k-Means method disqualified them as predictive classifiers. We conclude that PNN is a very effective tool for predicting 5–year overall survival in cervical cancer patients treated with radical hysterectomy.

## Abbreviations

Acc: Accuracy
AUROC: Area under receiver operating characteristic curve
BMI: Body mass index
FIGO: The International federation of gynecology and obstetrics
GEP: Gene expression programming
MLP: Multilayer perceptron
PNN: Probabilistic neural network
RBFNN: Radial basis function neural network
Sen: Sensitivity
Spe: Specificity
SVM: Support vector machines
